# Supplementary material for: Association between oral microbiome and seven types of cancers in East Asian population: a two-sample Mendelian randomization analysis
Source: Front Mol Biosci. 2023 Nov 21;10:1327893. doi: 10.3389/fmolb.2023.1327893 (PMC10702768; doi:10.3389/fmolb.2023.1327893)
Supplement: Supplementary file 1 [file DataSheet1.ZIP › Supplementary Materials/MR plots for tongue/tongue═╝/Pancreatic cancer/╥╚╧┘░⌐_╔╕╤í╜ß╣√/pheno.3013_to_pancreatic cancer_leave_one_out.pdf]

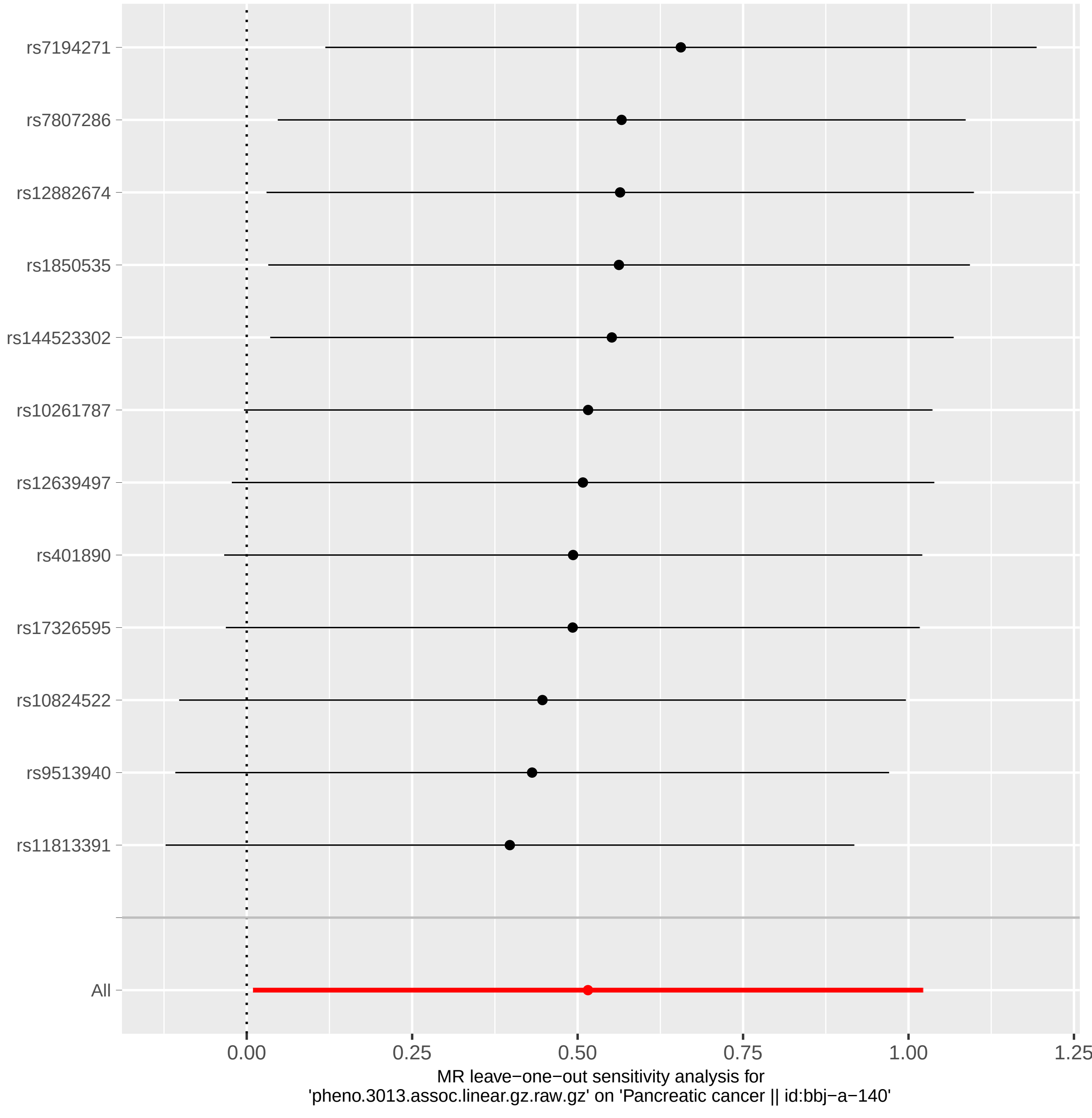
rs7194271
rs7807286
rs12882674
rs1850535
rs144523302
rs10261787
rs12639497
rs401890
rs17326595
rs10824522
rs9513940
rs11813391
All
0.00
0.25
0.50
0.75
1.00
1.25
MR leave−one−out sensitivity analysis for
'pheno.3013.assoc.linear.gz.raw.gz' on 'Pancreatic cancer || id:bbj−a−140'
